# Prevalence, country-specific prescribing patterns and determinants of benzodiazepine use in community-residing older adults in 7 European countries

**DOI:** 10.1186/s12877-024-04742-7

**Published:** 2024-03-07

**Authors:** Anna Lukačišinová, Jindra Reissigová, Maja Ortner-Hadžiabdić, Jovana Brkic, Betul Okuyan, Daisy Volmer, Ivana Tadić, Pilar Modamio, Eduardo L. Mariño, Konstantine Tachkov, Rosa Liperotti, Graziano Onder, Harriet Finne-Soveri, Hein van Hout, Elizabeth P. Howard, Daniela Fialová

**Affiliations:** 1 Department of Social and Clinical Pharmacy - Research Group “Ageing, Polypharmacy and Changes in the Therapeutic Value of Drugs in the Aged”, Faculty of Pharmacy in Hradec Králové, Charles University, Heyrovského 1203, Hradec Králové, 500 05 Czech Republic; 2 Department of Statistical Modelling, Institute of Computer Science of the Czech Academy of Sciences, Prague, Czech Republic; 3 Center for Applied Pharmacy, Faculty of Pharmacy and Biochemistry, University of Zagreb, Zagreb, Croatia; 4 Faculty of Pharmacy, University of Belgrade, Belgrade, Serbia; 5 Department of Clinical Pharmacy, Faculty of Pharmacy, Marmara University, Istanbul, Turkey; 6 Faculty of Medicine, Institute of Pharmacy, University of Tartu, Tartu, Estonia; 7 Clinical Pharmacy and Pharmaceutical Care Unit, Department of Pharmacy and Pharmaceutical Technology, and Physical Chemistry, Faculty of Pharmacy and Food Sciences, University of Barcelona, Barcelona, Spain; 8 Faculty of Pharmacy, Medical University of Sofia, Sofia, Bulgaria; 9 Fondazione Policlinico Universitario A. Gemelli IRCCS, Rome, Italy; 10 Università Cattolica del Sacro Cuore, Rome, Italy; 11 Finnish Institute for Health and Welfare, Helsinki, Finland; 12 Department of General Practice, Amsterdam Public Health Research Institute, Amsterdam University Medical Center, Vrije Universiteit, Amsterdam, The Netherlands; 13 Connell School of Nursing, Boston College, Chestnut Hill, MA USA; 14 The Hinda and Arthur Marcus Institute for Aging Research (The Marcus Institute), Hebrew Senior Life, Boston, MA USA; 15 Department of Geriatrics and Gerontology, 1st Faculty of Medicine in Prague, Charles University, Prague, Czech Republic

**Keywords:** Benzodiazepines, Community-dwelling older adults, Europe, Geriatric dosing, Geriatric length of therapy

## Abstract

**Background:**

The use of benzodiazepines (BZDs) in older population is often accompanied by drug-related complications. Inappropriate BZD use significantly alters older adults’ clinical and functional status. This study compares the prevalence, prescribing patterns and factors associated with BZD use in community-dwelling older patients in 7 European countries.

**Methods:**

International, cross-sectional study was conducted in community-dwelling older adults (65 +) in the Czech Republic, Serbia, Estonia, Bulgaria, Croatia, Turkey, and Spain between Feb2019 and Mar2020. Structured and standardized questionnaire based on interRAI assessment scales was applied. Logistic regression was used to evaluate factors associated with BZD use.

**Results:**

Out of 2,865 older patients (mean age 73.2 years ± 6.8, 61.2% women) 14.9% were BZD users. The highest prevalence of BZD use was identified in Croatia (35.5%), Spain (33.5%) and Serbia (31.3%). The most frequently prescribed BZDs were diazepam (27.9% of 426 BZD users), alprazolam (23.7%), bromazepam (22.8%) and lorazepam (16.7%). Independent factors associated with BZD use were female gender (OR 1.58, 95%CI 1.19–2.10), hyperpolypharmacy (OR 1.97, 95%CI 1.22–3.16), anxiety (OR 4.26, 95%CI 2.86–6.38), sleeping problems (OR 4.47, 95%CI 3.38–5.92), depression (OR 1.95, 95%CI 1.29–2.95), repetitive anxious complaints (OR 1.77, 95%CI 1.29–2.42), problems with syncope (OR 1.78, 95%CI 1.03–3.06), and loss of appetite (OR 0.60, 95%CI 0.38–0.94). In comparison to Croatia, residing in other countries was associated with lower odds of BZD use (ORs varied from 0.49 (95%CI 0.32–0.75) in Spain to 0.01 (95%CI 0.00–0.03) in Turkey), excluding Serbia (OR 1.11, 95%CI 0.79–1.56).

**Conclusions:**

Despite well-known negative effects, BZDs are still frequently prescribed in older outpatient population in European countries. Principles of safer geriatric prescribing and effective deprescribing strategies should be individually applied in older BZD users.

**Supplementary Information:**

The online version contains supplementary material available at 10.1186/s12877-024-04742-7.

## Introduction

Benzodiazepines (BZDs) are largely prescribed in many countries in different populations and care settings [1–5]. Increasing trend in consumption of BZDs worldwide is alarming [6]. By their low per-unit price, high volume sells, inexpensive manufacturing and minimal or no research and development costs, BZDs sales present a significant profit for pharmaceutical companies [7]. BZDs are indicated in various medical conditions such as anxiety, insomnia, panic attacks, epilepsy, muscle spasms and pre-surgical stress [1]. However, due to their well-known adverse effects, regulation of BZD use has been considered important in numerous countries [2–4].

As confirmed by previous studies, the prevalence of BZDs prescription is higher in older adults [5], which is mostly a consequence of accumulation of their long-term use with higher age and conversion of high proportion of new users to long-term users [8, 9]. The risk–benefit ratio of BZD use remains questionable, particularly in the older population. Adverse drug events including number of geriatric syndromes such as falls, fractures, functional and cognitive decline, psychomotor sedation, orthostasis and delirium associated with use of BZDs are well known [10–12]. Additionally, strong evidence of high risk of drug dependence associated with BZDs should not be overlooked [13]. Studies show that the risk of BZD dependence increases after 3 months of use by 20% and after 12 months use by 50% [14]. Moreover, potentially inappropriate prescription of BZDs in older adults was found to be linked with significantly higher health care costs [15].

Over the last decades, several studies reporting utilization of BZDs in older population in different countries have been published. However, diversities in study methodology, data collection and population characteristics make their comparisons challenging. In the light of above-mentioned scientific evidence, the objective of this study was to determine and compare the prevalence and prescribing patterns of BZD use in older community-dwelling patients in European countries. We focused also on description of factors influencing use of these medications that may help to better target future rational deprescribing strategies in older population.

## Methodology

### Study design

This was an international, cross-sectional study of 2,865 community-dwelling patients 65 + years old assessed in 7 European countries: the Czech Republic (*N* = 450), Serbia (*N* = 460), Estonia (*N* = 311), Bulgaria (*N* = 543), Croatia (*N* = 391), Turkey (*N* = 450) and Spain (*N* = 260). Data were collected between February 2019 and March 2020 as a part of the EuroAgeism H2020 ESR7 project. The sample size was determined based on the aim of the EuroAgeism H2020 study (to estimate the prevalence of potentially inappropriate medication (PIMs) use in participating countries). The magnitude of the prevalence has been reported to vary across countries, or to be unknown. Assuming a prevalence of 50% for PIM use in Central and Eastern EU countries, a minimum sample size of 385 patients in each country was confirmed to be involved in the study to reach a confidence level of 95% that the real prevalence is detected within ± 5% of the surveyed value. To account for known heterogeneity of PIMs and BZD use across regions, patients were planned to be assessed in 3 regionally different bigger cities/regions (about 150 patients per region). Exceptions were noted for Spain where due to study constrains during the COVID-19 period data were collected only in one region (the city of Barcelona, Catalonia). The other exception was Estonia, where due to lower population sizes at regional study sites, data were collected from 4 country regions. Total sample size in Estonia was adjusted to the number of older adults in population and a total of 311 older patients were finally recruited.

### Inclusion and exclusion criteria

Every community-dwelling older patient 65 + years old visiting pharmacies in stable health status (no intensive care, no acute worsening of health status requiring hospitalization or emergency department visit in the last 14 days, no palliative or terminal care, with an expected survival of more than 12 months) were included in this study. Patients with severe dementia (equal to Mini Mental State Examination score bellow 10), severe communication, hearing, or speech impairment (unable to respond to research questions or unable to give informed consent) were excluded. The refusal rate at all study sites did not exceed 10%. The sample was convenient, not randomly selected and was not intended to be representative of all older community-residing adults in the country.

### Data collection

A structured, standardized, and piloted research questionnaire, enabling a comprehensive geriatric assessment was used for data collection. It comprised patient-related characteristics, e.g., socio-demographic, clinical status, physical and cognitive status information, medical diagnoses, symptoms, signs, patient medication information and selected laboratory tests results (if available). Depression and anxiety and their severity were documented either from diagnoses and symptoms patient reported as part of the patient assessment or calculated using an adjusted Depression Rating Scale (DRS) [16] from other assessed characteristics in the questionnaire. The original DRS [16] was adjusted according to available data, and it was calculated by summing seven items (1. Feeling of sadness, 2. Persistent anger with self or others, 3. Expressions, including non-verbal, of what appear to be unrealistic fears, 4. Repetitive health complaints, 5. Repetitive anxious complaints/concerns (non-health related), 6. Sad, pained or worried facial expressions, 7. Crying, tearfulness) and scored 0 = problems were not exhibited, score 1 = problems exhibited, at least once in last 30 days, score 2 = problems exhibited up to 5 days a week, and score 3 = problems exhibited daily or almost daily (6–7 days a week). Before summing items, level 3 was recoded to 2. The DRS score ranges from 0 (no symptoms of depression) to 14 (all depressive symptoms present daily or almost daily, corresponding to severe depression). Clinically relevant depression is equal to score 3 or higher [16].

Morbidity (number of diagnoses) was defined as a sum of all identified diagnoses out of 68 diagnoses surveyed. When identifying number of diagnoses, we excluded pneumonia which was not collected in Bulgaria, and lower number of other blood cells which was not collected in Estonia.

All medications patients took in the last 7 days prior to the assessment, including medications on as needed bases *(pro re nata – PRN)*, were recorded including the drug name, Anatomical Therapeutic and Chemical (ATC) code [17], formulation, dosage, frequency, and route of administration.

### Outcomes

Primary outcomes of this study were: (1) BZD prevalence; (2) patterns of BZD use; (3) BDZ daily dose and length of use; and (4) factors associated with BZD use.

To capture all possible BZDs, all existing BZD ATC codes available at WHO international classification [17] were analysed (see Additional Table 1). The overall daily dose used by each BZD user was calculated as the sum of single doses applied during the day (morning, afternoon, evening, bedtime doses). In case the dose of BZD was not reported or was unavailable, it was referred as “unknown” (see Table 2). Data on length of BZD use (see Fig. 2) reported as “unknown” refer to situation when the length of use was not reported due to either missing values or due to inability of the patient to recall this information. However these two possible options were not distinguished in the data.

### Statistical analysis

The differences in the categorical variables across countries were evaluated using the chi-square test if all expected counts were at least 5, otherwise, the Fisher’s exact test was used. Prevalence of BZD users was expressed as a percentage with 95% confidence interval calculated using the Clopper–Pearson method. Direct age-standardized BZD prevalence was calculated using the 2013 European Standard Population (65–69, 70–74, 75–79, 80–84, ≥ 85) to adjust for differences in the age structures of the compared countries. Logistic regression was performed to explore variables related to use of BZDs namely: country, age, gender, number of medications (without BZDs), number of diagnoses, DRS, items on mood changes (presence of persistent anger, expressing unrealistic fears, repetitive health complaints, repetitive anxious complaints, reporting being sad, anxious or worried, reporting crying or tearfulness), self-reported mood (reporting having little interest or pleasure, reporting being anxious, restless or uneasy behaviour, reporting being sad, depressed or hopeless), reported diagnoses (dementia, depression, anxiety disorder, sleeping problems, panic disorder), and symptoms (chronic pain, shortness of breath, loss of appetite, vertigo, unsteady gait, hypotension, syncope, bradycardia, history of falls in the last year). Both the likelihood ratio test and the Akaike information criterion were used for selection of significant predictors. Standard logistic regression diagnostics (residual analysis, the Cook's distance) was performed to detect outliers and highly influential observations. The generalized variance inflation factor was used for detecting multicollinearity. The accuracy of the logistic regression was measured by the omnibus goodness of fit (GOF) test, the McFadden's R-squared and the C-statistics*.* Results of logistic regression were expressed as odds ratio (ORs) with their 95% (profile likelihood) confidence intervals (CIs).

In addition to logistic regression, multilevel (also called mixed) logistic regression was used for exploring the three-level clustered data structure: patient (level 1) nested within region/cities (level 2) nested within countries (level 3). Country and city/region were estimated as random intercepts and the other potential predictors of the BZD use as fixed effects. In short, the model selection was based on similar statistics described above. Random effect distributions were examined graphically. The adjusted intraclass correlation coefficient (ICC) was calculated to measure the degree of clustering within countries and regions/cities.

The level of statistical significance was set at *p* < 0.05. All data were analysed using R software, version 4.1.1.

### Ethical approval

Ethical approval was obtained from the ethical committees of all participating countries according to local regulations. Only patients who signed informed consent were included in the study. Patients were free to decline participation at any time during the study. Data were collected and stored under specific codes with an assurance of anonymity and confidentiality. Study forms and datasets were secured by the Central Information System of the Main Coordinating Centre according to rules of GDPR, H2020 European projects and data protection policy of the Charles University, Czech Republic.

## Results

The sample of 2,865 patients had a mean age (± standard deviation [SD]) of 73.2 ± 6.8 years and the majority were women (61.2%). The mean ± SD number of medications used was 4.7 ± 3.0, with majority (93.5%) of patients taking up to 9 different medications. Details on main characteristics of the study population are given in Table 1.

**Table 1:** Main characteristics of the study sample^a^

	**Total**	**BG**	**CZ**	**EE**	**ES**	**HR**	**RS**	**TR**	***P*-value**^**f**^
	***N* = 2,865 (%)**	***N* = 543 (%)**	***N* = 450 (%)**	***N* = 311 (%)**	***N* = 260 (%)**	***N* = 391 (%)**	***N* = 460 (%)**	***N* = 450 (%)**	
**Age (years)**									
65–74	1,801 (63.1)	365 (68.1)	330 (73.3)	185 (59.5)	110 (42.3)	217 (56.2)	292 (63.5)	302 (67.1)	< 0.001
75–84	813 (28.5)	131 (24.4)	96 (21.3)	96 (30.9)	101 (38.8)	135 (35.0)	126 (27.4)	128 (28.4)	
> = 85	239 (8.4)	40 (7.5)	24 (5.3)	30 (9.6)	49 (18.8)	34 (8.8)	42 (9.1)	20 (4.4)	
**Gender**									
Male	1,105 (38.8)	189 (36.1)	169 (37.6)	92 (29.6)	92 (35.4)	143 (36.6)	191 (41.5)	229 (50.9)	< 0.001
Female	1,740 (61.2)	334 (63.9)	281 (62.4)	219 (70.4)	168 (64.6)	248 (63.4)	269 (58.5)	221 (49.1)	
**Number of medications**^**b**^									
0–4	1,508 (52.6)	373 (68.7)	323 (71.8)	146 (46.9)	124 (47.7)	146 (37.3)	198 (43.0)	198 (44.0)	< 0.001
5–9	1,171 (40.9)	163 (30.0)	111 (24.7)	140 (45.0)	108 (41.5)	204(52.2)	234 (50.9)	211 (46.9)	
> = 10	186 (6.5)	7 (1.3)	16 (3.6)	25 (8.0)	28 (10.8)	41 (10.5)	28 (6.1)	41 (9.1)	
**Number of diagnoses**^**c**^									
0–3	1,361 (48.4)	312 (58.9)	266 (59.2)	196 (63.2)	69 (26.5)	96 (27.0)	226 (49.3)	196 (43.7)	< 0.001
> = 4	1,450 (51.6)	218 (41.1)	183 (40.8)	114 (36.8)	191 (73.5)	259 (73.0)	232 (50.7)	253 (56.3)	
**DRS**^**d**^									
0	1,397 (49.6)	206 (41.2)	318 (70.7)	96 (30.9)	129 (49.6)	162 (42.2)	262 (57.0)	224 (49.9)	< 0.001
1–2	583 (20.7)	108 (21.6)	89 (19.8)	99 (31.8)	31 (11.9)	88 (22.9)	69 (15.0)	99 (22.0)	
> = 3	834 (29.6)	186 (37.2)	43 (9.6)	116 (37.3)	100 (38.5)	134 (34.9)	129 (28.0)	126 (28.1)	
**Dementia**	66 (2.3)	12 (2.2)	4 (0.9)	1 (0.3)	1 (0.4)	12 (3.1)	27 (5.9)	9 (2.0)	< 0.001
**Depression**	262 (9.2)	41 (7.6)	33 (7.3)	18 (5.8)	50 (19.2)	37 (9.5)	41 (8.9)	42 (9.3)	< 0.001
**Anxiety disorder**	238 (8.3)	22 (4.1)	23 (5.1)	8 (2.6)	49 (18.8)	73 (18.8)	29 (6.3)	34 (7.6)	< 0.001
**Sleeping problem**	557 (19.5)	26 (4.8)	79 (17.6)	36 (11.6)	131 (50.4)	130 (33.5)	112 (24.3)	43 (9.6)	< 0.001
**Panic disorder**	32 (1.1)	9 (1.7)	1 (0.2)	0 (0.0)	7 (2.7)	1 (0.3)	5 (1.1)	9 (2.0)	< 0.001
**History of falls**^**e**^	349 (15.1)	-	39 (8.7)	69 (22.2)	28 (10.8)	70 (18.0)	58 (12.6)	85 (18.9)	< 0.001
**Number of BZD used**									
0	2,439 (85.1)	527 (97.1)	439 (97.6)	285 (91.6)	173 (66.5)	252 (64.5)	316 (68.7)	447 (99.3)	< 0.001
1	406 (14.2)	16 (2.9)	10 (2.2)	25 (8.0)	83 (31.9)	132 (33.8)	137 (29.8)	3 (0.7)	
2	19 (0.7)	0 (0.0)	1 (0.2)	1 (0.3)	4 (1.5)	7 (1.8)	6 (1.3)	0 (0.0)	
3	1 (0.0)	0 (0.0)	0 (0.0)	0 (0.0)	0 (0.0)	0 (0.0)	1 (0.2)	0 (0.0)	

*BG* Bulgaria, *CZ* Czech Republic, *EE* Estonia, *ES* Spain, *HR* Croatia, *RS* Serbia, *TR* Turkey

^a^Percentages calculated from non-missing value (missing values < 2% in Total)

^b^ Number of medications was calculated using the ATC codes, supplements and over the counter medications were not included

^c^ Number of diagnoses – altogether 68 diagnoses were surveyed in this study. Pneumonia was exclude from the sum of diagnoses, as it was not collected in Bulgaria. Lower number of other blood cells was excluded from the sum of diagnoses, as it was not collected in Estonia

^d^ Depression scale [16] – The original Depression scale (DRS) [16] was adjusted according to available data, and it was calculated by summing seven items (1. Feeling of sadness, 2. Persistent anger with self or others, 3. Expressions, including non-verbal, of what appear to be unrealistic fears, 4. Repetitive health complaints, 5. Repetitive anxious complaints/concerns (non-health related), 6. Sad, pained or worried facial expressions, 7. Crying, tearfulness) and scored 0 = problems were not exhibited, score 1 = problems exhibited, at least once in last 30 days, score 2 = problems exhibited up to 5 days a week, and score 3 = problems exhibited daily or almost daily (6–7 days a week). Before summing items, level 3 was recoded to 2. The DRS score ranges from 0 (no symptoms of depression) to 14 (all depressive symptoms present daily or almost daily, relevant to severe depression). Clinically relevant depression is equal to score 3 or higher

^e^ History of at least one fall in the last year; Data on history of falls was not available in Bulgaria

^f^ *p*-value of the chi-square test or the Fisher’s exact test

### Prevalence and patterns of BZD use across countries

**Fig. 1:**
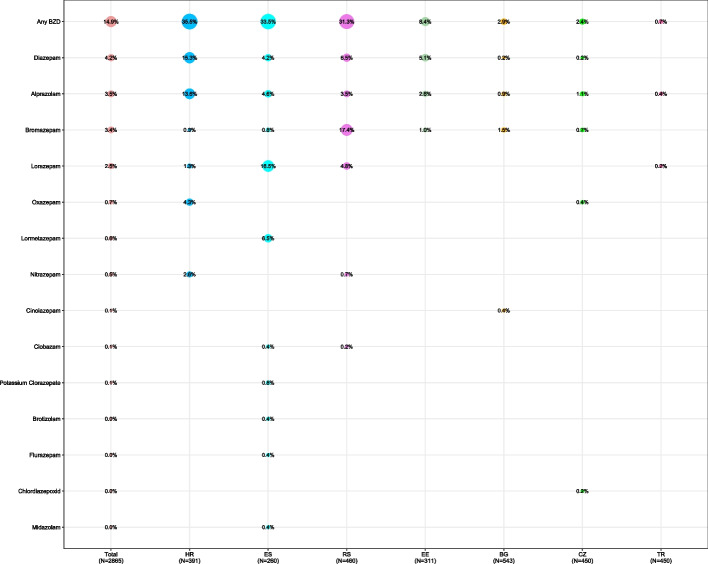
Prevalence of individual BZDs across participating countries^a^. ^a^BG – Bulgaria, CZ – Czech Republic, EE – Estonia, ES – Spain, HR – Croatia, RS – Serbia, TR – Turkey; Only prevalence of non-null occurrence of BZD displayed; BZD combinations not presented

Overall, there were 426 (14.9%) patients using at least 1 BZD. Figure 1 shows the prevalence and distribution of individual BZD users across participating countries, with the highest prevalence in Croatia (35.5% BZD users out of 391 patients) and the lowest in Turkey (0.7% BZD users out of 450 patients). There were total of 447 BZDs used in the sample. 20 patients were identified as users of multiple BZDs (users of more than 1 BZD), representing 4.7% of all 426 BZD users. The number of patients using different combinations of BZDs is given in Table 1, with additional details in Additional file Table 2.

The four most frequently prescribed BZDs were diazepam, alprazolam, bromazepam and lorazepam representing 26.6%, 22.6%, 21.7%, and 15.9% of all BZDs used (*N* = 447); respectively. In three countries with the highest BZD prevalence, there was always one dominant BZD used (% calculated from BZDs users in a particular country): diazepam (43.2% of 139) in Croatia; lorazepam (49.4% of 87) in Spain; and bromazepam (55.6% of 144) in Serbia. For details on patterns of BZD use see Additional file Figs. 1 and 2.

### Doses and length of BZD use

Recommended dose for older population [18–20] was exceeded in users of all four most frequently prescribed BZDs (Table 2). Dose of lorazepam higher than 1.0 mg per day (the recommended maximum geriatric daily dose is 1 mg [18]) was used by 33.8% of 71 lorazepam users and dose of diazepam higher than 5 mg (the recommended maximum geriatric daily dose is 5 mg [18]) was used by 28.6% of 119 diazepam users. In 24.8% of 101 alprazolam users the dose was higher than 0.75 mg (the recommended maximum geriatric daily dose is 0.75 mg [18]) and 5.2% of 97 bromazepam users were prescribed overall daily dose higher than 3 mg (the recommended maximum geriatric daily dose is 3 mg [18]) (Table 2).

**Table 2:** Spectrum of doses of the 4 most frequently used BZDs in the study^a^

Diazepam			Alprazolam			Bromazepam			Lorazepam		
**Daily dose**^**b**^ **[mg]**	N	%	**Daily dose**^**b**^ **[mg]**	N	%	**Daily dose**^**b**^ **[mg]**	N	%	**Daily dose**^**b**^ **[mg]**	N	%
2	3	2.5	0.0625	1	1.0	1.5	18	17.5	0.5	4	5.6
4	4	3.4	0.25	13	12.9	2	1	1.0	1	40	56.3
5	40	33.6	0.5	35	34.7	2.25	1	1.0	**1.25**	**1**	**1.4**
**7.5**	**1**	**0.8**	0.75	2	2.0	3	59	60.8	**1.5**	**1**	**1.4**
**10**	**27**	**22.7**	**1**	**14**	**13.9**	**4.5**	**1**	**1**	**2**	**3**	**4.2**
**15**	**3**	**2.5**	**1.5**	**6**	**5.9**	**6**	**3**	**3.1**	**2.5**	**15**	**21.1**
**19**	**1**	**0.8**	**2**	**3**	**3.0**	**9**	**1**	**1.0**	**4**	**2**	**2.8**
**20**	**1**	**0.8**	**3**	**2**	**2.0**				**5**	**2**	**2.8**
**30**	**1**	**0.8**									
PRN^c^	37	31.1	PRN^c^	25	24.8	PRN^c^	12	12.4	PRN^c^	3	4.2
Unknown^d^	1	0.8	Unknown^d^	0	0.0	Unknown^d^	1	2.1	Unknown^d^	0	0.0
Total	119	100	Total	101	100	Total	97	100	Total	71	100.0

^a^ Parts highlighted in bold represent doses that are higher than usual recommended geriatric doses for older population:

5 mg for diazepam [18], 0.75 mg for alprazolam [18], 3 mg for bromazepam [18] and 1 mg for lorazepam [18]

^b^ Daily dose was calculated as dose strength of 1 unit * number of units given per day (morning + afternoon + evening + bedtime)

^c^ PRN – pro re nata – used on “as needed” basis, there were no available data on daily dose in this dosage regime

^d^ Unknown data on dose – information on dose unavailable

When the number or patients with specified doses was used as a denominator to calculate the percentage of users of doses exceeding geriatric limits, we obtained these findings: higher dose was used in 42.0% of 81 diazepam users, 32.9% of 76 alprazolam users, 6.0% of 84 bromazepam users, and 35.3% of 68 lorazepam users. The mean daily doses ± SD (median) were 7.6 mg ± 4.4 mg (5.0 mg) for diazepam, 0.8 mg ± 0.6mg (0.5 mg) for alprazolam, 2.9 mg ± 1.1 mg (3.0 mg) for bromazepam and 1.6 mg ± 1.0 mg (1.0 mg) for lorazepam.

**Fig. 2:**
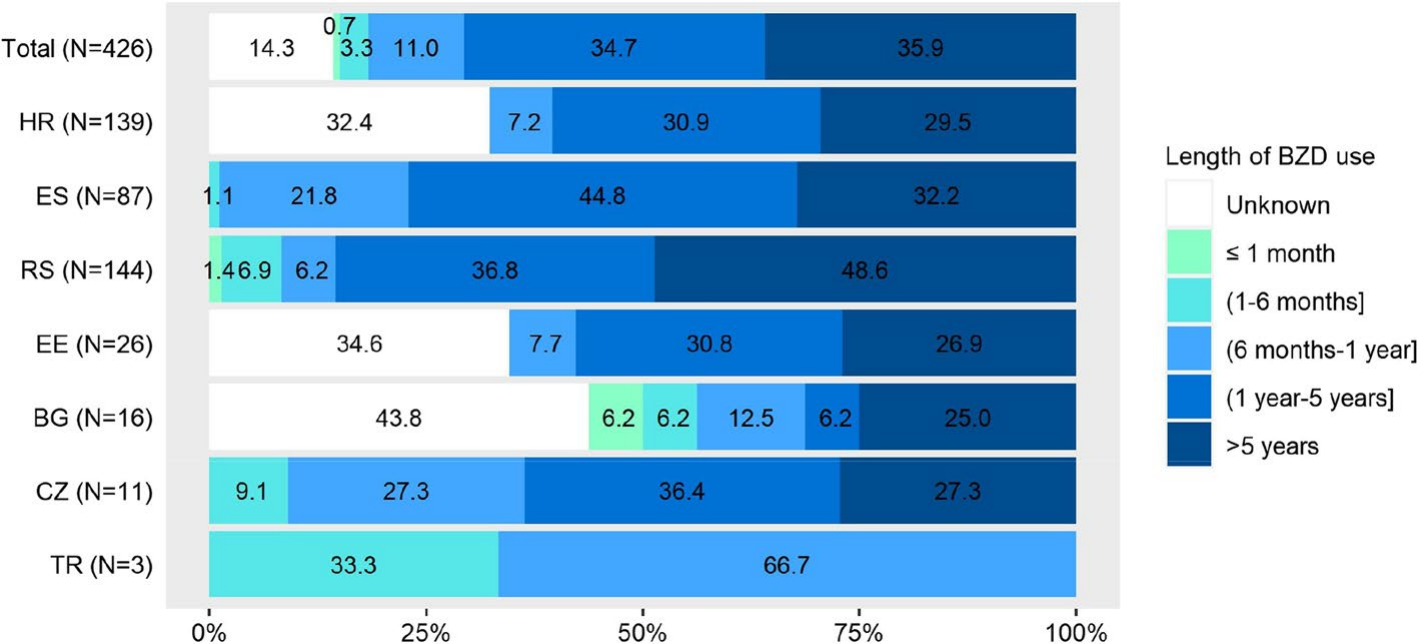
Length of BZD use and cross-country differences^a^. ^a^BG – Bulgaria, CZ – Czech Republic, EE – Estonia, ES – Spain, HR – Croatia, RS – Serbia, TR – Turkey; N – Number of BZDs used in the country; Percentages were calculated using a denominator including missing values. When missing values were excluded from denominator (*N* = 365) the corresponding results were: in total sample of BZD users 0.8% of patients used BZDs for period of less than 1 month, 3.8% for 1 – 6 months, 12.9% for 6 months – 1 year, 40.5% for 1 – 5 years, and 41.9% for more than 5 years

Overall, 70.7% of 426 BZD users used BZDs for more than 1 year, while 35.9% for more than 5 years. In 14.3% of patients the length of use was unknown. Only 0.7% of BZD users used BZDs (at the time of data collection) for less than 1 month. Details on the length of BZD use are given in Fig. 2.

### Factors associated with BZD use

Basic characteristics of BZD users and non-users are shown in Additional file Table 3. According to multiple logistic regression, the use of BZDs was statistically significantly associated with female gender, hyperpolypharmacy (10 + medications, without BZDs), presence of anxiety disorder, sleeping problems, depression, repetitive anxious complains, loss of appetite, and syncope. The country of residence was also independent factor associated with BZD use—compared to Croatia (ref.), patients in Spain, Estonia, Bulgaria, the Czech Republic and Turkey had significantly lower odds of using BZDs. For details see Table 3. The presented model has good properties (the omnibus GOF test: *p* = 0.296; the C statistics = 0.900; the McFadden's R-squared = 37%). When we applied multilevel logistic regression model (mixed model – see Methodology section) to the data, the same predictors (defined as fixed effects) as those in Table 3 were significant. The ICC at the country-level was 39.5% and at the city/region within country level 2.7%. The multilevel model results are not shown here (to our knowledge, so far, little research has been conducted regarding sample size of three-level logistic regression models).

**Table 3:** Results of the multiple logistic regression model

	**BZD users** ***N* = 423 (%)**	**BZD non-users** ***N* = 2384 (%)**	**Adjusted** **OR**^**b**^	**95% LCI**	**95% UCI**	***p*-value**
**Country**						
Croatia	136 (32.2)	244 (10.2)	1			
Serbia	144 (34.0)	316 (13.3)	1.11	0.79	1.56	0.543
Spain	87 (20.6)	173 (7.3)	0.49	0.32	0.75	0.001
Estonia	26 (6.1)	284 (11.9)	0.23	0.14	0.37	< 0.001
Bulgaria	16 (3.8)	481 (20.2)	0.08	0.04	0.14	< 0.001
Czech Republic	11 (2.6)	439 (18.4)	0.05	0.02	0.09	< 0.001
Turkey	3 (0.7)	447 (18.8)	0.01	0.00	0.03	< 0.001
**Gender**						
Male	116 (27.4)	979 (41.1)	1			
Female	307 (72.6)	1405 (58.9)	1.58	1.19	2.1	0.002
**Number of medications**^**a**^						
< 10	372 (87.9)	2,268 (95.1)	1			
> = 10	51 (12.1)	116 (4.9)	1.97	1.22	3.16	0.005
**Depression**						
Not present	324 (76.6)	2,224 (93.3)	1			
Present	99 (23.4)	160 (6.7)	1.95	1.29	2.95	0.002
**Anxiety disorder**						
Not present	297 (70.2)	2,275 (95.4)	1			
Present	126 (29.8)	109 (4.6)	4.26	2.86	6.38	< 0.001
**Sleeping problem**						
Not present	178 (42.1)	2,074 (87.0)	1			
Present	245 (57.9)	310 (13.0)	4.47	3.38	5.92	< 0.001
**Repetitive anxious complaints**						
Not exhibited	288 (68.1)	1,972 (82.7)	1			
Exhibited	135 (31.9)	412 (17.3)	1.77	1.29	2.42	< 0.001
**Loss of appetite**						
No	377 (89.1)	2,159 (90.6)	1			
Yes	46 (10.9)	225 (9.4)	0.6	0.38	0.94	0.027
**Syncope**						
No	384 (90.8)	2,310 (96.9)	1			
Yes	39 (9.2)	74 (3.1)	1.78	1.03	3.06	0.038

^a^Number of medications does not include BZDs

^b^Adjusted for all variables presented in the Table 3

## Discussion

The overall prevalence of BZD use in 7 European countries involved in our study was almost 15%, with Croatia (36%), Spain (34%) and Serbia (31%) having the highest prevalence. It is of importance to note that in all countries included in this study, BZD availability on the pharmaceutical market is bound to medical prescription. Country of residence was in our study recognized as an independent factor associated with BZD use. Variability in overall BZD prevalence and diverse spectrum of BZDs used in different countries indicates for country-specific differences in prescribing habits and availabilities of BZDs on the pharmaceutical markets, as well as different prescribing policies, and other social, cultural, and behavioral factors that play an important role in BZD use.

### Prevalence and patterns of BZD use across countries

In Croatia in 2016, BZDs were prescribed in 23%, 19%, and 16% of patients in 60–69, 70–79 and 80 + years age groups; respectively [21]. It is assumed that war in Croatia during 1990s was the initiator of higher consumption of BZDs [21]. Reports on consumption of BZDs in Croatia show constant increase of BZD use [22] and lately coincidence with Covid-19 pandemic [23]. According to the recent national data, diazepam, alprazolam and oxazepam were the most frequently used BZDs in Croatia [21], which corresponds with our study. All BZDs available on the pharmaceutical market in Croatia are listed as essential or supplementary medications covered fully or partially by health insurance by the Croatian Health Insurance Fund (CHIF) [21]. It is important to mention, that oxazepam is listed as supplementary medication and is not fully cowered by the CHIF. Diazepam, on the other hand, is on the list of essential medications with full coverage. This might be one of the reasons for higher prevalence of diazepam use in our Croatian sample compared to oxazepam, although scientific evidence favours use of the latter in older patients. The only prescription limitation implemented by CHIF was prescription of a drug for a maximum of 30 days and a maximum of 2 packages of the same drug per prescription [24]. Currently, there are no specific national guidelines for BZD use in older population and national scientific literature offers a various range of recommendations on the length of BZD use depending on dosage, type of BZD and diagnosis [21].

In Serbia, data for general population show constantly increasing trend in BZD prescription. First noted in 1980s, in former Yugoslavia, and repeatedly confirmed in 1990s, with sudden surge in 1999, the year of NATO air strikes to the country [25], BZD consumption continued raising in 2000s [26]. In our study both bromazepam and diazepam were most frequently used BZDs in Serbian sample. Bromazepam users were found almost solely in Serbia (83% of all bromazepam users). Bromazepam, diazepam and lorazepam were the most commonly used BZDs in Serbia in general population between 2014 and 2018 [27]. A study of patients 65 + years old admitted to the emergency ward due to BZD poisoning showed bromazepam and diazepam as the most frequently prescribed BZDs [28]. The only restriction of BZD prescription in Serbia is the maximum of 30 days stockpile [27]. As a matter of fact, no specific restriction of length together with possibility of re-prescribing without need of specialist consultation allow use of BZDs in Serbia for longer period than recommended [27].

In Spain, the Spanish Agency of Medicines and Sanitary Devices stipulated for BZDs, that their information sheet should state the recommended duration of treatment to be as short as possible and not exceeding 4 weeks for insomnia and 8 weeks for anxiety indications [29]. Moreover, the Spanish Society of Family and Community Medicine recommends short-term use of BZD just for alleviation of symptoms and for limited periods [30]. Nevertheless, our study found a prevalence of BZD use in Spanish population of almost 34%. Use of BZDs in Spain in older outpatients was reported to range from 14 to 38% in previous studies [31, 32]. A study of BZD users in Barcelona showed that 96% of BZDs were prescribed *off label* (defined as inappropriate dose, length of use and duplicity), while older patients were the most common group to be exposed to such *off-label* use [33]. In the study of BZD dependence in Spain outpatient population, 47% to 44% of older BZD users were BZD dependent [34]. Moreover, almost half of the population involved in the referred study were not given any medical advice about the risk of dependence [34]. In 2019, BZDs belonged to the 15 most frequently prescribed medications in Spain, accounting for retail value of about 98 million euros [35]. Our study showed that lorazepam was the most prevalent BZD in Spain, followed by lormetazepam, alprazolam and diazepam, which corresponds with recent national findings [35]. Moreover, lormetazepam was found to be the most frequently prescribed BZD in older patients admitted to the emergency ward due to falls [36]. A recent qualitative study of barriers and enablers for BZD (de)prescription revealed that the issue of high prevalence of BZD prescription in Spanish general population is strongly related to factors beyond purely clinical ones, such as lack of time and excessive workload, inadequate communication between doctors and patients, and extend of psychosocial skills of doctors [37].

In light of the above mentioned, the extent of BZD use in the older Croatian, Serbian and Spanish community-dwelling patients identified in our study is alarming. Specific guidelines and effective policy measures for reduction of BZD use should be applied.

In our study, the prevalence of BZD use was low in Bulgaria, the Czech Republic, and Turkey. There is a scarcity of data on the use of BZDs in Bulgaria. The latest national study estimated odds of 2.1 for PIMs prescription (including BZDs) in older population with an expected 31% of those leading to a drug related problem [38]. According to the referred national study there are no national guidelines established for any kind of medication review in the older population or collection of PIMs [38]. For the Czech Republic, studies show the prevalence of BZD use about 20% [39] in older nursing home residents. However, educational strategies in the past decades significantly contributed to reduction in prescribing of BZDs in populations of older adults in other settings of care, particularly in community-dwelling older patients, who present significantly different population regarding cognitive, functional, and overall health status. On the other hand there are no current national guidelines or health care policy regulations in the Czech Republic that would regulate use of BZDs in particular. Prescription of BZDs in the Czech Republic is not bound to any specialization or prescribing conditions. In Turkey, BZDs were prescribed at least once a year in 4% of older patients with psychiatric disorders [40]. The low prescription rates of BZDs can be associated with low preference of BZD use by physicians due to their addictive potential and unwillingness of patients to use BZDs commonly perceived as “heavy drugs” [40]. The fact that BZDs are listed as controlled substances and their prescription is strictly regulated in Turkey [41], can also contribute to low prevalence of BZD use found in our study.

### Doses and length of BZD use

Maximal doses for older adults recommended by international PIMs criteria [18–20] were exceeded in all four most frequently prescribed BZDs in our study. Studies confirm that about 14% of older population is prescribed BZDs in higher than recommended doses and suggest association between type of BZD used and propensity of risk of high dose intake [42, 43]. Regarding the appropriateness of BZD doses, the literature differs in cut-off points (i.e., amount of mg) and in methodology of dose calculation. In our study, a daily dose for each patient and each BZD was calculated separately without any further adjustments. This allows for examination of patterns of use of each BZD individually.

The usual maximum length of BZD therapy is set up to 1 month for treatment of insomnia, anxiety, and initiation of depression treatment [20]. In our study, 71% of 426 BZD users used these medications for more than 1 year, while 36% were users for more than 5 years. Only 1% of BZD users used BZDs for less than 1 month. Some studies show older patients being more likely to be long-term users and users of higher doses compared to younger patients [43, 44]. The length of BZD use in older long-term users was proved to be associated with higher risk of hospitalization/emergency department visits due to falls [45].

The length of BZD use accompanied by the proportion of patients taking higher doses in our study is alarming. Specific interventions are needed emphasizing early deprescribing strategies and intensifying other more rational, safer ways of treatment. Tools like specialized software might help in every day clinical practice to specifically monitor dosing, length of therapy, and risk factors of inappropriate drug treatment in older adults. Although this study did not aim to assess appropriateness of BZD use, the results show the prevalence of long-term BZD use remains high. Up to date a vast number of guidelines and recommendations to reduce inappropriate use of BZDs was developed [46–48]. Numerous screening tools such as Beers criteria [19], STOPP/START criteria [20] as well as educational programs or deprescribing tools such as Eliminating Medication Through Patient Ownership of End Results (EMPOWER) study [49], Reducing Use of Sedatives (RedUSe) project [50] and Halting Antipsychotic Use in Long-Term (HALT) study [51] have been introduced in previous decades. Interventions to lower BZD use in older patients mainly consist of establishing a specific deprescribing protocol, education of physicians, pharmacists, nurses and patients with focus on non-pharmacological prevention and management of symptoms for witch BZDs were originally prescribed (such as anxiety, insomnia, etc.).

In most of the countries included in our study there is some kind of BZD prescription regulation present, suggesting there must be other factors influencing the magnitude of BZD prescription. A recent qualitative systematic review of healthcare professionals’, patients’ and family caregivers’ attitudes towards the use of psychotropic medication (including BZDs) in older people, showed that we are dealing with multidimensional problem [52]. While being aware of existence of international guidelines of BZD use, healthcare professionals identified barriers to following them on individual, team and organizational levels [52]. The situation with alternative non-pharmacological approaches seems to be complicated as well. Some studies revealed lack of time, training, funding and accessibility as well as resistance of patients to accept psychological interventions, to be main barriers in non-pharmacological strategies incorporation [52].

### Factors associated with BZD use

Factors associated with BZD use described in our study might play a critical role in identifying patients at risk and need of specific interventions to reduce extensive inappropriate use of BZDs.

In our study use of BZDs was statistically significantly associated with female gender which is in line with findings in some other studies [53]. Higher age, in our study, was significantly associated with BZD use only in the simple (univariable) model, however the adjusted association did not remain statistically significant. This is in contrary to other recent findings suggesting advanced age (85 + years) to be a protective factor of prescribing potentially inappropriate polypharmacy in older population [54]. Even though the overall research evidence points to awareness of prescribing of PIMs in older patients, findings of our study may suggest that the population of community-dwelling older patients is different compared to other specific populations such as nursing home residents, acute hospital in-patients, etc. Differences in functional, cognitive, and overall health status may lead to different approaches in prescription of PIMs in these unique populations of geriatric patients.

The presence of anxiety disorder, depression, and repetitive anxious complains were positively associated with BZD use in our study. Studies suggest that older patients in primary care are at higher risk of inappropriate BZD prescription coupled with underutilization of antidepressants in geriatric depression [55, 56]. Association of insomnia with BZD use in our study corresponds with general knowledge of BZD prescription in older patients for insomnia and anxiety [57], despite their minimal efficacy in these indications [19]. While the BZD prescription in these indications might be seen as appropriate in middle age population, safer therapeutic options should be considered for older patients. Several interdependent factors leading to difficulties in change of BZD prescription patterns were described. These include for instance physicians’ insufficient recognition of adverse effects, lack of skills and training in BZD tapering management, as well as patients’ resistance to change their medication regime, limited availability of psychotherapists, and absence of regular specialist medication reviews [58].

In our study, BZD users had significantly higher odds of presence of syncope compared to BZD non-users. BZDs are associated with greater systolic blood pressure reduction after an active stand-up [59]. It is of importance to emphasize that syncope may be consequent adverse drug event of BZD use, responsible also for falls, fractures and intracranial haemorrhage [60], resulting in hospitalization, reduced mobility, decline in functional status, and higher risk of long-term care facilities admission [61]. BZDs are considered one of the major fall-risk-increasing drugs, as they increase the risk of falls by approximately 50% [62]. Annual costs of treatment for BZD related fall injuries in the European Union were estimated up to 1.8 billion Euros [63].

Our study showed BZD users had lower odds of reporting loss of appetite. The hyperphagic effect of BZDs was connected to their direct effect on appetite mechanism and enhanced responsiveness to sweet, palatable taste [64]. Although these findings are linked to research on animal models, interestingly loss of appetite is sometimes identified as one of the symptoms of BZD withdrawal [65]. On the other hand, loss of appetite can be also linked to presence of anxiety and successful anxiety treatment with BZDs might come hand in hand with appetite regulation.

### Strengths and limitations

To the best of our knowledge, this study delivers the first and broadest comparisons of BZD prevalence and prescribing patterns in population of community-dwelling older patients across European countries. Using the same comprehensive methodology at all study sites enabled us to make meaningful comparisons in participating countries.

The main limitation of our study is that the sample was not randomly selected. On the other hand, study sites were selected as general health care facilities- pharmacies, not providing services to specialized psychiatric health-care clinics. Country samples were not intended to be nationally representative, therefor the results of this study need to be interpreted with caution in regards to generalizability. The cross-sectional design of the study did not allow for comparison in a time-dependent manner or to identify causal time relationships between BZD use and factors used in multiple regression models. The focus of this study was to describe specifically BZD use, and as such, other drugs prescribed for similar indications and non-pharmacological treatment strategies were not analysed. The individual appropriateness of BZD use was not assessed, as this study did not focus on implicit medication reviews. We did not control our analyses for all clinical comorbidities (except for number of diagnoses), however, in multiple regression model main comorbidities well known to be associated with BZD use were included.

## Conclusions

This study showed significant differences in BZD prevalence and prescribing patterns across several European countries, and it also documented very frequent long-term BZD use and frequent use of non-geriatric doses. Importance of clinical and non-clinical factors (prescribing habits, social, cultural, economic, and behavioural factors, etc.) and regulatory and policy interventions and their contribution to the current use of BZDs were discussed with respect to currently available national evidence.

It is of particular importance to further investigate factors contributing to unnecessary and inappropriate BZD use on national as well as European level, because they allow planning of relevant interventions helping to reduce BZD burden and support the future wellbeing of older population.

### Supplementary Information

**Additional file 1: Figure 1.** Differences in BZD pattern in BZD users across three countries with the highest prevalence of BZD use^a^.

**Additional file 2: Figure 2.** Crude prevalence of users of at least 1 BZD and distribution of individual BZDs across participating countries^a^.

**Additional file 3: Table 1.** List of ATC codes and drug names included into the analyses.

**Additional file 4: Table 2.** List of combinations of 2 or 3 different BZDs used at the same time^a^.

**Additional file 5: Table 3.** Differences in main characteristic between BZD users and BZD non-users^a^.

## Data Availability

The datasets used and/or analysed during the current study are available from the corresponding author upon reasonable request.
