# Supplementary material for: Prevalence, country-specific prescribing patterns and determinants of benzodiazepine use in community-residing older adults in 7 European countries
Source: BMC Geriatr. 2024 Mar 7;24:240. doi: 10.1186/s12877-024-04742-7 (PMC10921596; doi:10.1186/s12877-024-04742-7)
Supplement: Supplementary file 1 — Additional file 1: Figure 1. Differences in BZD pattern in BZD users across three countries with the highest prevalence of BZD usea. [file 12877_2024_4742_MOESM1_ESM.docx]

**Additional Figure 1.** Differences in BZD pattern in BZD users across three countries with the highest prevalence of BZD use^a^

^
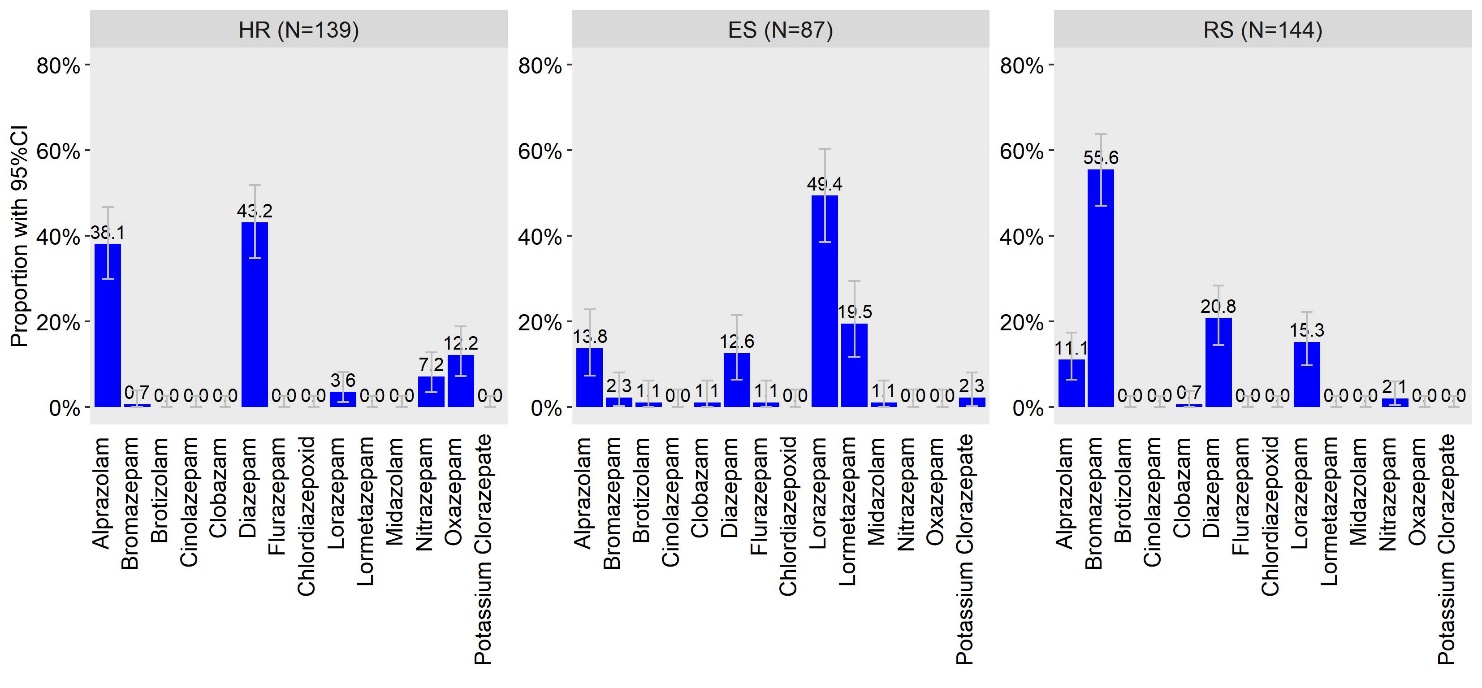
^

^a^*ES – Spain, HR – Croatia, RS – Serbia; N – Number of BZDs used in the country; Only countries with prevalence of BZD > 10% are showed*
