# Supplementary material for: Prevalence, country-specific prescribing patterns and determinants of benzodiazepine use in community-residing older adults in 7 European countries
Source: BMC Geriatr. 2024 Mar 7;24:240. doi: 10.1186/s12877-024-04742-7 (PMC10921596; doi:10.1186/s12877-024-04742-7)
Supplement: Supplementary file 2 — Additional file 2: Figure 2. Crude prevalence of users of at least 1 BZD and distribution of individual BZDs across participating countriesa. [file 12877_2024_4742_MOESM2_ESM.docx]

**Additional Figure 2.** Crude prevalence of users of at least 1 BZD and distribution of individual BZDs across participating countries^a^

^
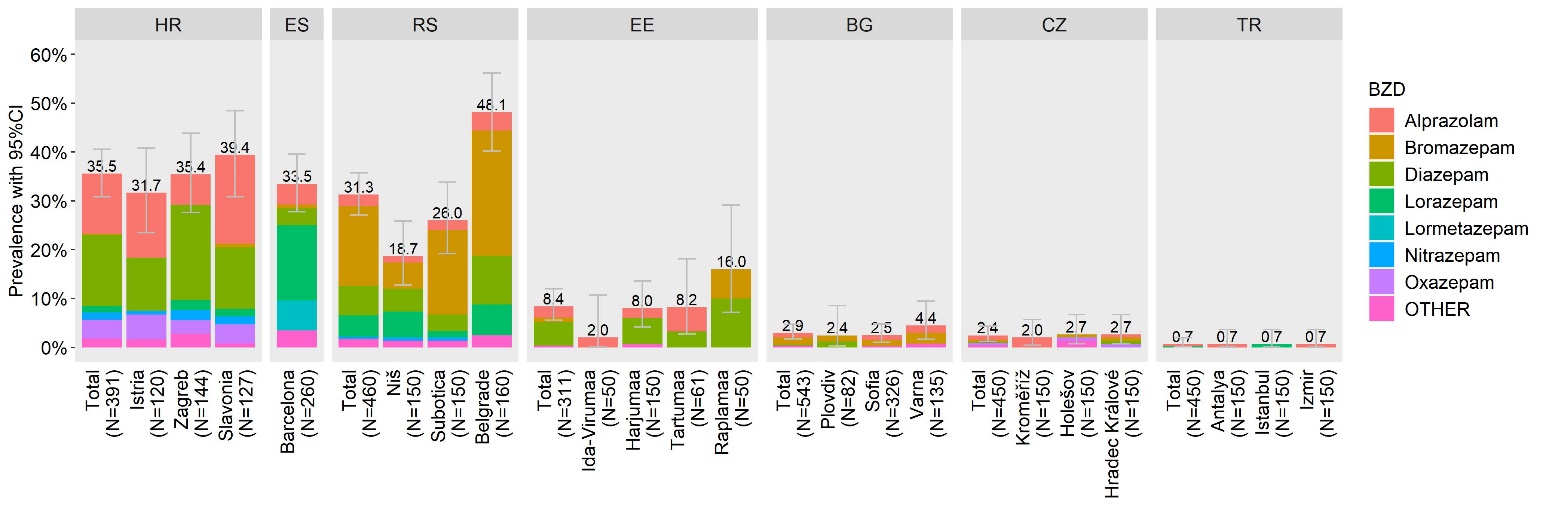
^

^a^*BG – Bulgaria, CZ – Czech Republic, EE – Estonia, ES – Spain, HR – Croatia, RS – Serbia, TR – Turkey; OTHER – includes different BZD combination;*

*Directly age-standardised prevalence (using European Standard Population 2013): HR: 36.2% (95%CI 30.4%;43.1%), ES: 33.1% (95%CI 26.2%;41.4%), RS: 32.1% (95%CI 26.9%;38.1%), EE: 8.8% (95%CI 5.8%;13.1%), CZ: 3.5% (95%CI 1.6%;6.8%), BG: 3.1% (95%CI 1.7%;5.3%), TR: 0.7% (95%CI 0.1%;3.2%)*
