# Supplementary material for: Prevalence, country-specific prescribing patterns and determinants of benzodiazepine use in community-residing older adults in 7 European countries
Source: BMC Geriatr. 2024 Mar 7;24:240. doi: 10.1186/s12877-024-04742-7 (PMC10921596; doi:10.1186/s12877-024-04742-7)
Supplement: Supplementary file 3 — Additional file 3: Table 1. List of ATC codes and drug names included into the analyses. [file 12877_2024_4742_MOESM3_ESM.docx]

**Additional Table 1.** List of ATC codes and drug names included into the analyses.

| **ATC code** | **Drug name** | **ATC code** | **Drug name** |
| --- | --- | --- | --- |
| *Benzodiazepine anxiolytics* | | *Benzodiazepine hypnotics* | |
| N05BA01 | Diazepam | N05CD01 | Flurazepam |
| N05BA02 | Chlordiazepoxid | N05CD02 | Nitrazepam |
| N05BA03 | Medazepam | N05CD03 | Flunitrazepam |
| N05BA04 | Oxazepam | N05CD04 | Estazolam |
| N05BA05 | Potassium Clorazepate | N05CD05 | Triazolam |
| N05BA06 | Lorazepam | N05CD06 | Lormetazepam |
| N05BA07 | Adinazolam | N05CD07 | Temazepam |
| N05BA08 | Bromazepam | N05CD08 | Midazolam |
| N05BA09 | Clobazam | N05CD09 | Brotizolam |
| N05BA10 | Ketazolam | N05CD10 | Quazepam |
| N05BA11 | Prazepam | N05CD11 | Loprazolam |
| N05BA12 | Alprazolam | N05CD12 | Doxefazepam |
| N05BA13 | Halazepam | N05CD13 | Cinolazepam |
| N05BA14 | Pinazepam |  |  |
| N05BA15 | Camazepam |  |  |
| N05BA16 | Nordazepam |  |  |
| N05BA17 | Fludiazepam |  |  |
| N05BA18 | Ethyl Loflazepate |  |  |
| N05BA19 | Etizolam |  |  |
| N05BA21 | Clotiazepam |  |  |
| N05BA22 | Cloxazolam |  |  |
| N05BA23 | Tofisopam |  |  |
| N05BA56 | Lorazepam, combinations |  |  |
