# Supplementary material for: Prevalence, country-specific prescribing patterns and determinants of benzodiazepine use in community-residing older adults in 7 European countries
Source: BMC Geriatr. 2024 Mar 7;24:240. doi: 10.1186/s12877-024-04742-7 (PMC10921596; doi:10.1186/s12877-024-04742-7)
Supplement: Supplementary file 4 — Additional file 4: Table 2. List of combinations of 2 or 3 different BZDs used at the same timea. [file 12877_2024_4742_MOESM4_ESM.docx]

**Additional Table 2.** List of combinations of 2 or 3 different BZDs used at the same time *^a^*

|  | BG | CZ | EE | ES | HR | RS | TR | Total |
| --- | --- | --- | --- | --- | --- | --- | --- | --- |
|  | N=543 | N=450 | N=311 | N=260 | N=391 | N=460 | N=450 | N=2865 |
| **Different ATC combination** |  |  |  |  |  |  |  |  |
| Alprazolam + Bromazepam | 0 | 1 | 0 | 0 | 0 | 2 | 0 | 3 |
| Alprazolam + Bromazepam + Diazepam | 0 | 0 | 0 | 0 | 0 | 1 | 0 | 1 |
| Alprazolam + Diazepam | 0 | 0 | 1 | 0 | 2 | 0 | 0 | 3 |
| Alprazolam + Lorazepam | 0 | 0 | 0 | 0 | 0 | 2 | 0 | 2 |
| Alprazolam + Lormetazepam | 0 | 0 | 0 | 1 | 0 | 0 | 0 | 1 |
| Alprazolam + Nitrazepam | 0 | 0 | 0 | 0 | 2 | 0 | 0 | 2 |
| Alprazolam + Oxazepam | 0 | 0 | 0 | 0 | 1 | 0 | 0 | 1 |
| Bromazepam + Diazepam | 0 | 0 | 0 | 0 | 0 | 1 | 0 | 1 |
| Bromazepam + Nitrazepam | 0 | 0 | 0 | 0 | 0 | 1 | 0 | 1 |
| Diazepam + Lorazepam | 0 | 0 | 0 | 2 | 0 | 0 | 0 | 2 |
| Diazepam + Nitrazepam | 0 | 0 | 0 | 0 | 1 | 0 | 0 | 1 |
| Lorazepam + Midazolam | 0 | 0 | 0 | 1 | 0 | 0 | 0 | 1 |
| Nitrazepam + Oxazepam | 0 | 0 | 0 | 0 | 1 | 0 | 0 | 1 |
| Total | 0 | 1 | 1 | 4 | 7 | 7 | 0 | 20 |
| **Same ATC combinations** |  |  |  |  |  |  |  |  |
| Alprazolam + Alprazolam | 0 | 0 | 0 | 0 | 2^b^ | 0 | 0 | 2 |
| Bromazepam + Bromazepam | 0 | 0 | 0 | 0 | 0 | 1^c^ | 0 | 1 |
| Diazepam + Diazepam | 0 | 0 | 1^d^ | 0 | 0 | 0 | 0 | 1 |
| Total | 0 | 0 | 1 | 0 | 2 | 1 | 0 | 0 |

*^a^ BG – Bulgaria, CZ – Czech Republic, EE – Estonia, ES – Spain, HR – Croatia, RS – Serbia, TR – Turkey*

*^b^ One patient had a combination of alprazolam 1mg (1 dose at bedtime, indication anxiety) + alprazolam 0.25mg (1 morning and 1 afternoon dose, indication anxiety); One patient had a combination of alprazolam 1mg (1 morning dose, indication anxiety) + alprazolam 1mg prolonged form (1 afternoon and 1 evening dose, indication anxiety)*

*^c^ Combination of bromazepam 3mg (PRN, indication anxiety) + bromazepam 3mg (PRN, indication anxiety)*

*^d^ Combination of diazepam 5mg (1 evening dose, indication anxiety) + diazepam 10mg (1 dose at bedtime, indication insomnia)*
