# Supplementary material for: Prevalence, country-specific prescribing patterns and determinants of benzodiazepine use in community-residing older adults in 7 European countries
Source: BMC Geriatr. 2024 Mar 7;24:240. doi: 10.1186/s12877-024-04742-7 (PMC10921596; doi:10.1186/s12877-024-04742-7)
Supplement: Supplementary file 5 — Additional file 5: Table 3. Differences in main characteristic between BZD users and BZD non-usersa. [file 12877_2024_4742_MOESM5_ESM.docx]

**Additional Table 3.** Differences in main characteristic between BZD users and BZD non-users^a^

|  | **BZD users** | | **BZD non-users** | | **Crude  OR** | **95% LCI** | **95% UCI** | **p-value** |
| --- | --- | --- | --- | --- | --- | --- | --- | --- |
|  | **N=426 (%)** | | **N=2439 (%)** | |  |  |  |  |
| **Country** |  |  |  |  |  |  |  |  |
| Croatia | 139 | (32.6%) | 252 | (10.3%) | 1 |  |  |  |
| Serbia | 144 | (33.8%) | 316 | (13.0%) | 0.83 | 0.62 | 1.10 | 0.190 |
| Spain | 87 | (20.4%) | 173 | (7.1%) | 0.91 | 0.65 | 1.27 | 0.584 |
| Estonia | 26 | (6.1%) | 285 | (11.7%) | 0.17 | 0.10 | 0.26 | <0.001 |
| Bulgaria | 16 | (3.8%) | 527 | (21.6%) | 0.06 | 0.03 | 0.09 | <0.001 |
| Czech Republic | 11 | (2.6%) | 439 | (18.0%) | 0.05 | 0.02 | 0.08 | <0.001 |
| Turkey | 3 | (0.7%) | 447 | (18.3%) | 0.01 | 0.00 | 0.03 | <0.001 |
| **Age (years)** |  |  |  |  |  |  |  |  |
| 65-74 | 223 | (52.5%) | 1578 | (65.0%) | 1 |  |  |  |
| 75-84 | 143 | (33.6%) | 670 | (27.6%) | 1.51 | 1.20 | 1.90 | <0.001 |
| >=85 | 59 | (13.9%) | 180 | (7.4%) | 2.32 | 1.66 | 3.20 | <0.001 |
| **Gender** |  |  |  |  |  |  |  |  |
| Male | 116 | (27.2%) | 989 | (40.9%) | 1 |  |  |  |
| Female | 310 | (72.8%) | 1430 | (59.1%) | 1.85 | 1.48 | 2.33 | <0.001 |
| **Number of medications^b^** |  |  |  |  |  |  |  |  |
| 0-4 | 186 | (43.7%) | 1397 | (57.3%) | 1 |  |  |  |
| 5-9 | 188 | (44.1%) | 926 | (38.0%) | 1.52 | 1.22 | 1.9 | <0.001 |
| >=10 | 52 | (12.2%) | 116 | (4.8%) | 3.37 | 2.33 | 4.81 | <0.001 |
| **Number of diagnoses^c^** |  |  |  |  |  |  |  |  |
| 0-3 | 98 | (23.8%) | 1263 | (52.6%) | 1 |  |  |  |
| >=4 | 313 | (76.2%) | 1137 | (47.4%) | 3.55 | 2.80 | 4.53 | <0.001 |
| **DRS^d^** |  |  |  |  |  |  |  |  |
| 0 | 153 | (36.3%) | 1244 | (52.0%) | 1 |  |  |  |
| 1-2 | 85 | (20.2%) | 498 | (20.8%) | 1.39 | 1.04 | 1.84 | 0.024 |
| >=3 | 183 | (43.5%) | 651 | (27.2%) | 2.29 | 1.81 | 2.89 | <0.001 |
| **Mood^e^** |  |  |  |  |  |  |  |  |
| **Persistent anger** |  |  |  |  |  |  |  |  |
| Not exhibited | 306 | (72.0%) | 1943 | (80.6%) | 1 |  |  |  |
| Exhibited, but not daily | 95 | (22.4%) | 413 | (17.1%) | 1.46 | 1.13 | 1.88 | 0.003 |
| Exhibited, daily | 24 | (5.6%) | 54 | (2.2%) | 2.82 | 1.69 | 4.58 | <0.001 |
| **Expressions of unrealistic fears** |  |  |  |  |  |  |  |  |
| Not exhibited | 297 | (69.9%) | 2010 | (83.4%) | 1 |  |  |  |
| Exhibited, but not daily | 104 | (24.5%) | 344 | (14.3%) | 2.05 | 1.59 | 2.62 | <0.001 |
| Exhibited, daily | 24 | (5.6%) | 56 | (2.3%) | 2.9 | 1.74 | 4.69 | <0.001 |
| **Repetitive health complaints** |  |  |  |  |  |  |  |  |
| Not exhibited | 273 | (64.5%) | 1849 | (76.7%) | 1 |  |  |  |
| Exhibited, but not daily | 119 | (28.1%) | 432 | (17.9%) | 1.87 | 1.46 | 2.37 | <0.001 |
| Exhibited, daily | 31 | (7.3%) | 129 | (5.4%) | 1.63 | 1.06 | 2.43 | 0.021 |
| **Repetitive anxious complaints** |  |  |  |  |  |  |  |  |
| Not exhibited | 289 | (68.0%) | 1993 | (82.7%) | 1 |  |  |  |
| Exhibited, but not daily | 111 | (26.1%) | 340 | (14.1%) | 2.25 | 1.75 | 2.88 | <0.001 |
| Exhibited, daily | 25 | (5.9%) | 77 | (3.2%) | 2.24 | 1.38 | 3.52 | 0.001 |
| **Sad, pained or worried** |  |  |  |  |  |  |  |  |
| Not exhibited | 268 | (63.2%) | 1841 | (76.4%) | 1 |  |  |  |
| Exhibited, but not daily | 129 | (30.4%) | 479 | (19.9%) | 1.85 | 1.46 | 2.33 | <0.001 |
| Exhibited, daily | 27 | (6.4%) | 91 | (3.8%) | 2.04 | 1.28 | 3.15 | 0.002 |
| **Crying or tearfulness** |  |  |  |  |  |  |  |  |
| Not exhibited | 296 | (70.1%) | 1968 | (81.8%) | 1 |  |  |  |
| Exhibited, but not daily | 100 | (23.7%) | 378 | (15.7%) | 1.76 | 1.36 | 2.26 | <0.001 |
| Exhibited, daily | 26 | (6.2%) | 61 | (2.5%) | 2.83 | 1.74 | 4.51 | <0.001 |
| **Self-reported mood** |  | | | | | | | |
| **Little interest or pleasure** |  | | | | | | | |
| No | 313 | (73.6%) | 1752 | (72.8%) | 1 |  |  |  |
| Yes | 112 | (26.4%) | 653 | (27.2%) | 0.96 | 0.76 | 1.21 | 0.732 |
| **Anxious, restless or uneasy** |  | | | | | | | |
| No | 304 | (71.5%) | 1869 | (77.7%) | 1 |  |  |  |
| Yes | 121 | (28.5%) | 535 | (22.3%) | 1.39 | 1.10 | 1.75 | 0.005 |
| **Sad, depressed, or hopeless** |  | | | | | | | |
| No | 305 | (71.8%) | 1909 | (79.4%) | 1 |  |  |  |
| Yes | 120 | (28.2%) | 495 | (20.6%) | 1.52 | 1.20 | 1.91 | <0.001 |
| **Diagnosis** |  |  |  |  |  |  |  |  |
| **Dementia** |  | | | | | | | |
| Not present | 408 | (95.8%) | 2389 | (98.0%) | 1 |  |  |  |
| Present | 18 | (4.2%) | 48 | (2.0%) | 2.20 | 1.23 | 3.74 | 0.005 |
| **Depression** |  | | | | | | | |
| Not present | 327 | (76.8%) | 2274 | (93.3%) | 1 |  |  |  |
| Present | 99 | (23.2%) | 163 | (6.7%) | 4.22 | 3.20 | 5.55 | <0.001 |
| **Anxiety disorder** |  | | | | | | | |
| Not present | 298 | (70.0%) | 2326 | (95.5%) | 1 |  |  |  |
| Present | 128 | (30.0%) | 110 | (4.5%) | 9.08 | 6.86 | 12.06 | <0.001 |
| **Sleeping problem** |  | | | | | | | |
| Not present | 180 | (42.3%) | 2125 | (87.2%) | 1 |  |  |  |
| Present | 246 | (57.7%) | 311 | (12.8%) | 9.34 | 7.46 | 11.72 | <0.001 |
| **Panic disorder** |  | | | | | | | |
| Not present | 414 | (97.2%) | 2417 | (99.2%) | 1 |  |  |  |
| Present | 12 | (2.8%) | 20 | (0.8%) | 3.50 | 1.65 | 7.12 | 0.001 |
| **Symptoms** |  | | | | | | | |
| **Chronic Pain** |  | | | | | | | |
| No | 220 | (51.6%) | 1403 | (57.7%) | 1 |  |  |  |
| Yes | 206 | (48.4%) | 1029 | (42.3%) | 1.28 | 1.04 | 1.57 | 0.02 |
| **Shortness of breath** |  | | | | | | | |
| No | 304 | (71.5%) | 1901 | (78.0%) | 1 |  |  |  |
| Yes | 121 | (28.5%) | 537 | (22.0%) | 1.41 | 1.11 | 1.77 | 0.004 |
| **Loss of appetite** |  | | | | | | | |
| No | 380 | (89.2%) | 2206 | (90.6%) | 1 |  |  |  |
| Yes | 46 | (10.8%) | 230 | (9.4%) | 1.16 | 0.82 | 1.61 | 0.382 |
| **Vertigo** |  | | | | | | | |
| No | 308 | (72.5%) | 2007 | (82.3%) | 1 |  |  |  |
| Yes | 117 | (27.5%) | 431 | (17.7%) | 1.77 | 1.39 | 2.24 | <0.001 |
| **Unsteady gait** |  | | | | | | | |
| No | 302 | (71.1%) | 2060 | (84.5%) | 1 |  |  |  |
| Yes | 123 | (28.9%) | 378 | (15.5%) | 2.22 | 1.75 | 2.81 | <0.001 |
| **Hypotension** |  | | | | | | | |
| No | 354 | (83.3%) | 2132 | (87.6%) | 1 |  |  |  |
| Yes | 71 | (16.7%) | 303 | (12.4%) | 1.41 | 1.06 | 1.86 | 0.017 |
| **Syncope** |  | | | | | | | |
| No | 385 | (90.8%) | 2360 | (96.9%) | 1 |  |  |  |
| Yes | 39 | (9.2%) | 75 | (3.1%) | 3.19 | 2.12 | 4.73 | <0.001 |
| **Bradycardia** |  | | | | | | | |
| No | 402 | (95.0%) | 2346 | (96.3%) | 1 |  |  |  |
| Yes | 21 | (5.0%) | 90 | (3.7%) | 1.36 | 0.82 | 2.17 | 0.214 |
| **History of falls^f^** |  | | | | | | | |
| No | 328 | (80.4%) | 1641 | (85.9%) | 1 |  |  |  |
| Yes | 80 | (19.6%) | 269 | (14.1%) | 1.49 | 1.12 | 1.95 | 0.005 |

*^a^ Percentages calculated from non-missing values*

*^b^ Number of medications does not include BZDs*

*^c^ Number of diagnoses – altogether 68 diagnoses were surveyed in this study. Pneumonia was exclude from the sum of diagnoses, as it was not collected in Bulgaria. Lower number of other blood cells was exclude from the sum of diagnoses, as it was not collected in Estonia.*

*^d^ Depression scale^16^ – The original Depression scale (DRS)^16^ was adjusted according to available data, and it was calculated by summing seven items (1. Feeling of sadness, 2. Persistent anger with self or others, 3. Expressions, including non-verbal, of what appear to be unrealistic fears, 4. Repetitive health complaints, 5. Repetitive anxious complaints/concerns (non-health related), 6. Sad, pained or worried facial expressions, 7. Crying, tearfulness) and scored 0 = problems were not exhibited, score 1 = problems exhibited, at least once in last 30 days, score 2= problems exhibited up to 5 days a week and score, 3 = problems exhibited daily or almost daily (6-7 days a week). Before summing items, level 3 was recoded to 2. The DRS score ranges from 0 (no symptoms of depression) to 14 (all depressive symptoms present daily all almost daily, relevant to severe depression). Clinically relevant depression is equal to score 3 or higher. ^e^ Exhibited, but not daily = Exhibited, at least once in the last 30 days, and up to 5 days a week; Exhibited, daily = Exhibited daily or almost daily (6,7 days a week)*

*^f^ History of at least one fall in the last year, Data on history of falls were not available in Bulgaria*
